# Antibiotic prescription strategies and adverse outcome for uncomplicated lower respiratory tract infections: prospective cough complication cohort (3C) study

**DOI:** 10.1136/bmj.j2148

**Published:** 2017-05-23

**Authors:** Paul Little, Beth Stuart, Sue Smith, Matthew J Thompson, Kyle Knox, Ann van den Bruel, Mark Lown, Michael Moore, David Mant

**Affiliations:** 1 University of Southampton, Primary Care Medical Group, PCPS Unit, Aldermoor Health Centre, Southampton SO16 5ST, UK; 2 Nuffield Department of Primary Health Care Sciences, University of Oxford, Radcliffe Observatory Quarter, Oxford, UK; 3 University of Washington, Seattle WA, USA

## Abstract

**Objective** To assess the impact on adverse outcomes of different antibiotic prescribing strategies for lower respiratory tract infections in people aged 16 years or more.

**Design** Prospective cohort study.

**Setting** UK general practice.

**Participants** 28 883 patients with lower respiratory tract infection; symptoms, signs, and antibiotic prescribing strategies were recorded at the index consultation.

**Main outcome measures** The main outcomes were reconsultation with symptoms of lower respiratory tract infection in the 30 days after the index consultation, hospital admission, or death. Multivariable analysis controlled for an extensive list of variables related to the propensity to prescribe antibiotics and for clustering by doctor.

**Results** Of the 28 883 participants, 104 (0.4%) were referred to hospital for radiographic investigation or admission, or both on the day of the index consultation, or were admitted with cancer. Of the remaining 28 779, subsequent hospital admission or death occurred in 26/7332 (0.3%) after no antibiotic prescription, 156/17 628 (0.9%) after prescription for immediate antibiotics, and 14/3819 (0.4%) after a prescription for delayed antibiotics. Multivariable analysis documented no reduction in hospital admission and death after immediate antibiotics (multivariable risk ratio 1.06, 95% confidence interval 0.63 to 1.81, P=0.84) and a non-significant reduction with delayed antibiotics (0.81, 0.41 to 1.64, P=0.61). Reconsultation for new, worsening, or non-resolving symptoms was common (1443/7332 (19.7%), 4455/17 628 (25.3%), and 538/3819 (14.1%), respectively) and was significantly reduced by delayed antibiotics (multivariable risk ratio 0.64, 0.57 to 0.72, P<0.001) but not by immediate antibiotics (0.98, 0.90 to 1.07, P=0.66).

**Conclusion** Prescribing immediate antibiotics may not reduce subsequent hospital admission or death for young people and adults with uncomplicated lower respiratory tract infection, and such events are uncommon. If clinicians are considering antibiotics, a delayed prescription may be preferable since it is associated with a reduced number of reconsultations for worsening illness.

## Introduction

Acute uncomplicated respiratory tract infections are the commonest acute illnesses managed in primary care in developed countries, and substantial numbers of patients still receive antibiotic treatment.^1^ ^2^ ^3^ The GRACE (Genomics to combat Resistance against Antibiotics for Community acquired lower respiratory tract infection (LRTI) in Europe) trial in lower respiratory tract infection (n=2061) documented no clear benefit of antibiotics (hazard ratio for duration of “moderately bad” symptoms 1.06, 95% confidence interval 0.96 to 1.18),^4^ and the updated Cochrane review provided similar estimates.^5^ Prescribing antibiotics incurs costs, increases reconsultation for subsequent episodes, medicalises self limiting illness,^6^ ^7^ and promotes antibiotic resistance, which is dominated by prescribing of antibiotics in primary care.^8^ However, both clinicians and patients worry about more severe or prolonged illness and complications,^9^ and clinicians fear medicolegal consequences.^2^ ^10^ ^11^ ^12^ Evidence to reassure clinicians is, however, limited: the Cochrane review did not document the impact of antibiotics on complications, and in the GRACE cohorts hospital admission or death occurred in fewer than 1% of patients who consulted, therefore large studies are needed.

Trial data can be limited by external validity and substantially greater drug compliance compared with observational settings.^13^ ^14^ Thus observational studies provide important evidence to complement trial data, but conversely have the disadvantage of confounding by indication. Therefore the impact of antibiotics should be assessed using techniques to control for the propensity to prescribe.^15^ ^16^ ^17^

We report the findings of an adequately powered observational study of lower respiratory tract infection in primary care using prospectively collected clinical data to assess the impact of antibiotics on adverse outcomes such as reconsultations with non-resolving or new symptoms, hospital admission, or death.

## Methods

A structured proforma was used to document clinical presenting features and management at the index consultation. Review of medical records documented reconsultations with new or worsening illness, hospital admission, or death during the next 30 days.

The study took place in UK primary care. Participants were eligible if they were aged 16 years or more and had acute lower respiratory tract infection—defined as acute cough (new or worsening cough for three weeks or less) presenting as the main symptom and judged to be infective in origin by the doctor, consistent with the Cochrane review^18^ and previous studies.^4^ ^6^ ^19^ ^20^ ^21^ ^22^ ^23^ ^24^ We excluded patients who had other causes of acute cough (eg, heart failure, acid reflux, fibrosing alveolitis), were unable to consent (eg, severe mental illness), were immunocompromised, had previously presented with the same episode of illness, or had been included previously.

### Data collection

#### Clinical record form

Doctors completed a clinical record form in the consultation, documenting age, smoking history, duration of symptoms, nature of symptoms (dry or productive cough, shortness of breath, coryza, fever, chills or shivering, chest pain, headache, muscle aches, sleep disturbance, confusion, diarrhoea, sputum colour), examination results (respiratory rate, pulse, blood pressure, oxygen saturation, temperature, presence of wheeze, crepitations, or bronchial breathing), a rating of overall severity (visual analogue scale ranging from “well” to “very unwell”), and if antibiotics were prescribed.

#### Outcomes (review of clinical records at 30 days)

The main outcome measures were reconsultation in primary care or visit to an emergency department with progression of illness (ie, with non-resolution of symptoms or new symptoms or signs)^25^ in the 30 days after the index consultation, hospital admission, or death.

#### Other data

Comorbidities noted in the medical records were also documented. Lung comorbidity included asthma, chronic obstructive pulmonary disease, or history of other important lung disease requiring hospital investigation, and use of steroids or bronchodilators. Vaccination status (pneumococcal vaccination) was recorded.

Practice staff assessed the clinical records using a structured proforma.^26^ Most baseline data were captured by electronic data capture live during consultations, and the small proportion completed on paper was double entered. Data recorded on paper at follow-up review were double entered. The patient’s postcode was used to assign deprivation, based on the national deprivation index.

### Sample size

The target of 28 024 patients was designed to detect predictors of rare (1:200) outcomes, with an odds ratio of 3 (α=0.01) and 80% power, among both those given antibiotics and those not given antibiotics. We also wanted to assess whether antibiotics reduced the number of uncommon outcomes: we assumed an antibiotic prescribing rate of 50% and estimated that the cohort would have more than 80% power to detect a one third reduction of outcome after antibiotics (odds ratio 0.66) for outcomes occurring with a frequency of 1% or more.

### Statistical analysis

The primary analysis concerned those presenting initially with uncomplicated illness—that is, we excluded patients admitted to hospital on the day of the index consultation, admitted to hospital with cancer, and with pneumonia confirmed by radiography ordered on the day of the consultation. In the remaining cohort we assessed the impact of antibiotics using logistic regression, accounting for clustering by doctor and controlling for any potential confounder of the association between prescribing strategy and outcome. Levels of missing data were mostly low: 76% of participants had complete data and 17% were only missing data on oxygen saturation (see supplementary table). We assessed the possible change in the estimates if all the missing data were imputed with either all low values or all high values for oxygen saturation.

Covariates were dichotomised according to clinically meaningful cut-points. We assessed the impact of controlling for all potentially relevant clinical and demographic variables collected at first presentation and also generated a model when just controlling for significant covariates (from backward fitting of the regression model, retaining all variables with P≤0.20). Relative risks were produced directly as the output from a generalised linear model for the binomial family.^27^ We then performed an analysis using a stratified propensity score method,^15^ ^16^ ^17^ which involved stratifying participants by fifths into mutually exclusive subsets based on their estimated propensity score, which in this dataset achieved a good balance of covariates in each stratum, key to providing control of confounding. This enabled exploration of whether more rigorous control for confounding by indication would modify the estimates. We calculated each propensity score comparing each prescribing strategy with no initial offer of antibiotics (ie, immediate antibiotics with no antibiotics, and delayed antibiotics with no antibiotics).

### Patient involvement

Since doctors’ clinical behaviour was our key focus, working general practitioners were involved in the design of the clinical record form and in interpreting findings. Patients were involved in agreeing constituent studies of the overall programme; advising about the patient information leaflets, consent forms, clinical record form, and outcomes; and participating regularly in study management meetings.

## Results

The National Institute for Health Research Clinical Research Network invited doctors nationwide in the UK to participate. Patients were recruited in 522 general practices between October 2009 and April 2013 after informed consent had been obtained (see supplementary figure). The practice median index of multiple deprivation score was 20.8, similar to the national figure of 21.5 from Public Health England. Overall, 28 883 patients had baseline and follow-up data available and 28 779 (99.6%) had an initially uncomplicated illness at the index consultation. Of these, 7332 (25.5%) received no prescription for antibiotics, 17 628 (61.3%) received a prescription for immediate antibiotics, and 3819 (13.3%) received a prescription for delayed antibiotics (median advised delay 3 days, interquartile range 2-3 days) at the index consultation. There were no major differences in clinical characteristics between higher and lower recruiting practices (see appendix 1).

### Patient characteristics

Major differences were found between antibiotic prescribing groups for some variables, particularly assessment of severity and lung findings. Participants prescribed immediate antibiotics were more likely to be older, have major comorbidities, and report more shortness of breath, fever, or purulent sputum, and the doctor was more likely to record higher severity ratings, low oxygen saturation, and crepitations or wheeze (table 1). Participants given a prescription for delayed antibiotics were commonly intermediate for the signs and symptoms between immediate and no prescription groups.

**Table 1 tbl1:** Patient characteristics. Values are numbers (percentages)

Characteristics	No antibiotics	Immediate antibiotics	Delayed antibiotics
Age ≥60 years	2112/7332 (28.8)	7456/17 628 (42.3)	1275/3819 (33.4)
Female	4448/7331 (60.7)	10 334/17 625 (58.6)	2284/3818 (59.8)
Illness duration <7 days	3498/7332 (47.7)	8851/17 628 (50.2)	1609/3819 (42.1)
Received pneumovax <10 years	1031/7332 (14.1)	3642/17 628 (20.7)	600/3819 (15.7)
Ever smoked	3570/7227 (49.4)	9700/17 327 (56.0)	1848/3757 (49.2)
Any comorbidity	2696/7332 (36.8)	8782/17 628 (49.8)	1582/3819 (41.4)
Lung comorbidity	1470/7332 (20.1)	5112/17 628 (29.0)	855/3819 (22.4)
Taking steroids or bronchodilators	1263/7057 (17.9)	4513/16 767 (26.9)	735/3646 (20.2)
Living in top 10th deprivation area (most deprived)*	1491/7332 (20.3)	3594/17 628 (20.4)	654/3819 (17.1)
Symptoms:			
Shortness of breath	3874/7325 (52.9)	12 306/17 531 (70.2)	2268/3805 (59.6)
Fever	2139/7329 (29.2)	7371/17 570 (42.0)	1439/3813 (37.7)
Chills	1824/7328 (24.9)	6116/17 567 (34.8)	1183/3806 (31.1)
Chest pain	2574/7325 (35.1)	6624/17 574 (37.7)	1414/3809 (37.1)
Confusion	399/7331 (5.4)	1241/17 611 (7.1)	216/3819 (5.7)
Coryza	4011/7325 (54.8)	9446/17 552 (53.8)	2235/3805 (58.7)
Headache	3127/7326 (42.7)	8254/17 562 (47.0)	1841/3807 (48.4)
Muscle aches	2364/7327 (32.3)	6747/17 563 (38.4)	1360/3808 (35.7)
Diarrhoea	563/7330 (7.7)	1600/17 608 (9.1)	339/3816 (8.9)
Sputum: purulent	3594/7329 (49.0)	12 133/17 628 (68.8)	2460/3818 (64.4)
Sputum: bloody/rusty	159/7329 (2.2)	740/17 628 (4.2)	110/3818 (2.9)
Clinical examination:			
Severity assessment ≥5/10	840/7332 (11.5)	9926/17 628 (56.3)	1092/2819 (28.6)
Respiratory rate >24/min	440/7299 (6.0)	2166/17 557 (12.3)	264/3806 (6.9)
Temperature ≥37.8°C	198/7328 (2.7)	1295/17 612 (7.4)	150/3818 (3.9)
Pulse ≥100/min	480/7329 (6.6)	2021/17 620 (11.5)	287/3818 (7.5)
O_2_ saturation <95%	166/6180 (2.7)	1403/14 350 (9.8)	120/3161 (3.8)
SBP <90 mm Hg or DBP <60 mm Hg	634/7332 (8.7)	1307/17 628 (7.4)	247/3819 (6.5)
Crackles	175/7330 (2.4)	11 220/17 622 (63.7)	814/3819 (21.3)
Bronchial breathing	91/7329 (1.2)	1916/17 618 (10.9)	149/3819 (3.9)
Wheeze	585/7330 (8.0)	5908/17 620 (33.5)	555/3819 (14.5)

SBP=systolic blood pressure; DBP=diastolic blood pressure.

*Index of multiple deprivation: Office of National Statistics, 2007.

### Hospital admission or death after uncomplicated presentations

Subsequent hospital admission or death occurred in 26/7332 (0.3%) after no antibiotic prescription, 156/17628 (0.9%) after an immediate prescription, and 14/3819 (0.4%) after a delayed prescription. Respiratory infections accounted for the greatest number of hospital admissions (n=131) and deaths (n=7), but cardiovascular or cerebrovascular events (admissions n=19 and deaths n=8) also occurred, in line with previous evidence of outcomes after lower respiratory tract infection,^28^ as did other infections (n=12 and n=0) and a variety of other events such as collapse, dehydration, and acute renal failure (n=20 and n=4). Most hospital admissions were for respiratory infections: 18/26 (58%) after no antibiotic prescription, 102/142 (72%) after immediate antibiotics, and 11/14 (79%) after delayed antibiotics (table 2). No admitted participant had allergic reactions, but one had amoxicillin induced cholestasis.

**Table 2 tbl2:** Hospital admissions and death. Values are numbers (percentages)

Factors	No antibiotics (n=7332)			Immediate antibiotics (n=17 628)				Delayed antibiotics (n=3819)	
	Admitted, no death	Admitted, death		Admitted, no death	Admitted, death	Death, not admitted		Admitted, no deaths	Admitted, death
LRTI or URTI	18 (0.25)	0 (0.00)		98 (0.56)	4 (0.02)	3 (0.02)		11 (0.29)	0 (0.00)
Cardiovascular or cerebrovascular event	0 (0.00)	1 (0.01)		15 (0.09)	2 (0.01)	5 (0.03)		1 (0.03)	0 (0.00)
Infections	2 (0.03)	0 (0.00)		8 (0.05)	1 (0.01)	0 (0.00)		1 (0.03)	0 (0.00)
Cancer	3 (0.04)	0 (0.00)		7 (0.04)	1 (0.01)	5 (0.03)		0 (0.00)	1 (0.03)
Other	5 (0.07)	0 (0.00)		10 (0.06)	4 (0.02)	0 (0.00)		1 (0.03)	0 (0.00)
No reason given	0 (0.00)	0 (0.00)		6 (0.03)	0 (0.00)	0 (0.00)		0 (0.00)	0 (0.00)

LRTI=lower respiratory tract infection; URTI=upper respiratory tract infection.

Data exclude those with pneumonia judged to be present clinically at the index consultation and those admitted on the same day as the index consultation; admissions and deaths were not exclusive categories.

When accounting for the propensity to prescribe there was no reduction in hospital admission and death after immediate antibiotics (multivariable risk ratio 1.06, 95% confidence interval 0.63 to 1.81, P=0.84) and a non-significant reduction with delayed antibiotics (0.81, 0.41 to 1.64, P=0.61; table 3). For immediate antibiotics, adequate balance was obtained for covariates (see appendix 1), and for delayed prescribing the balance for most but not all covariates improved.

**Table 3 tbl3:** Antibiotic prescribing strategy and hospital admission or death within 30 days

Antibiotic prescribing*	No (%) with no hospital admission or death	No (%) with hospital admission or death	Univariate risk ratio (95% CI)	P value	Risk ratio controlling for clustering and covariates (95% CI)	P value	Multivariable risk ratio controlling for clustering and significant covariates† (95% CI)	P value	Multivariable risk ratio adjusted using stratified propensity score (95% CI)	P value
None	7306/28 583 (25.6)	26/196 (13.3)	1.00		1.00		1.00		1.00	
Immediate	17472/28 583 (61.1)	156/196 (79.6)	2.35 (1.59 to 3.49)	<0.01	1.47 (0.80 to 2.72)	0.22	1.65 (0.97 to 2.80)	0.06	1.06 (0.63 to 1.81)	0.84
Delayed	3805/28 583 (13.3)	14/196 (7.1)	0.99 (0.53 to 1.85)	0.98	1.02 (0.51 to 2.05)	0.95	0.97 (0.49 to 1.92)	0.93	0.81 (0.41 to 1.64)	0.61

*Excluding those with pneumonia judged to be present clinically at the index consultation, those admitted on the day of index consultation, and those with cancer related admissions.

†Significant covariates: age ≥60 years, sex, any comorbidity, shortness of breath, chest pain, absence of coryza, respiratory rate >24/min, low blood pressure, severity score >4, pulse >100/min, oxygen saturation <95%, and temperature >37.8°C.

### Reconsultation with non-resolving or worsening symptoms

Reconsultation for new, worsening, or non-resolving symptoms was common: 1443/7332 (19.7%) for no antibiotics, 4455/17 628 (25.3%) for immediate antibiotics, and 538/3819 (14.1%) for delayed antibiotics. Delayed antibiotics were associated with a significant reduction in number of reconsultations (multivariable risk ratio 0.64, 0.57 to 0.72, P<0.01) but not immediate antibiotics (0.98, 0.90 to 1.07, P=0.97; table 4).

**Table 4 tbl4:** Antibiotic prescribing strategy and reconsultation within 30 days with same or worsening illness

Antibiotic prescribing*	No (%) with no reconsultation	No (%) with reconsultation	Univariate risk ratio (95% CI)	P value	Risk ratio controlling for clustering and all covariates (95% CI)	P value	Multivariable risk ratio controlling for clustering and significant covariates† (95% CI)	P value	Multivariable risk ratio adjusted using IPW propensity score (95% CI)	P value
None	5889/22 343 (26.4)	1443/6436 (22.4)	1.00		1.00		1.00		1.00	
Immediate	13173/22 343 (59.0)	4455/6436 (69.2)	1.28 (1.22 to 1.35)	<0.01	1.01 (0.93 to 1.10)	0.77	1.00 (0.92 to 1.08)	0.94	0.98 (0.90 to 1.07)	0.66
Delayed	3281/22 343 (14.7)	538/6436 (8.4)	0.72 (0.65 to 0.78)	<0.01	0.67 (0.60 to 0.76)	<0.01	0.67 (0.60 to 0.75)	<0.01	0.64 (0.57 to 0.72)	<0.01

IPW=inverse propensity weighting.

*Excluding those with pneumonia judged to be present clinically at the index consultation, those admitted on the day of index consultation, and those with cancer related admissions.

†Significant covariates: age ≥60 years, sex, pneumovax <10 years, use of inhaled steroid or bronchodilators, shortness of breath, fever, chest pain, absence of coryza, muscle aches, respiratory rate >24/min, wheeze, bronchial breathing, crackles, severity score >4, pulse >100/min, and oxygen saturation <95%.

### Potential bias due to missing data on oxygen saturation

The inferences were not changed either for reconsultations or for hospital admissions if missing values for oxygen saturation were imputed as either more than 95% saturation or less than 95% saturation (see supplementary table).

## Discussion

As far as we are aware this is one of the very few prospective clinical cohorts with sufficient power to address the impact of antibiotic prescribing strategies on subsequent major adverse outcomes. Even in a cohort of 30 000 participants, it was not possible to show that either immediate or delayed antibiotics were associated with a significant reduction in subsequent hospital admission or death. Non-resolving symptoms or progression of the illness with new symptoms was common, and a delayed prescription but not immediate antibiotics were associated with a significant reduction in reconsultations with such symptoms.

### Strengths and limitations of this study

This study has several main strengths: 1) sufficient power to assess rare adverse outcomes owing to the size of the cohort; 2) high follow-up; 3) assessment of similar variables to previous trials and observational datasets^4^ ^22^ ^29^ and inclusion of the key drivers identified from qualitative work^30^; 4) study of routine consultations in everyday primary care to provide a large generaliseable cohort, using a simple clinical proforma to facilitate recruitment; 5) a nationwide practice sample with deprivation scores similar to national figures; 6) the diagnosis of chest infections using clinical criteria similar to the Cochrane review^18^ and previous studies in primary care^4^ ^19^ ^20^ ^22^ ^29^ ^31^ ^32^; 7) clinical characteristics of included participants similar to those in previous trials and observational cohorts in primary care^4^ ^22^ ^23^ (approximately 20% with lung comorbidity, 70% with coloured sputum, previous illness duration of one week); 8) assessment of complications using a highly structured notes review for high reliability^26^; and 9) the rate of complications after presentation of a similar magnitude to previous studies using routine data.^2^

Limitations were that patients were recruited at the busiest times of year, and as with other studies of acute infection^14^ ^33^ documentation of the details of those not approached was poor owing to time pressures (since time pressures to recruit also meant time pressure to document non-recruitment). No training was given in measuring clinical signs and we had no mechanism for quality assuring diagnostic skills, but conversely this means that these results are more generaliseable to the routine clinical setting. Although nearly 20% of participants had missing data for oxygen saturation, inferences were not altered in the sensitivity analysis with imputed extreme values for missing data. Patients were not blind to their management and so knowledge of the receipt of antibiotics might have altered their threshold for consulting. Using stratified propensity scores resulted in important changes in the estimates: for immediate antibiotics adequate balance of covariates was achieved, making residual confounding less likely. For delayed prescribing the balance of covariates did not improve for all variables so we may have underestimated the impact of delayed prescribing on complications. We were also not powered to detect odds ratios greater than 0.66 for preventing complications. We did not measure reconsultations longer term, but there is reasonable trial evidence that both short and long term reattendance is reduced by delayed prescription.^6^ ^34^

### Comparison with previous research

We are aware of no large prospective cohort studies to have assessed the role of antibiotic prescribing strategies in everyday primary care practice for major adverse outcomes. Doctors are clearly using signs and symptoms to guide prescribing decisions: patients with more severe illness are more likely to receive a prescription for an antibiotic and those with intermediate severity delayed prescribing, which matches the behaviour of doctors in upper respiratory tract infections.^26^ The finding that subsequent hospital admission or death were not clearly reduced with delayed or immediate antibiotic prescribing could be due to inadequate control of confounding, but since we assessed a wide range of potentially confounding variables, a large impact of antibiotics seems unlikely. Even if we missed a small effect, subsequent major adverse events are uncommon, and in this cohort more than 60% of participants received an immediate antibiotic. If we use the lower limit of the 95% confidence interval of the risk ratio for immediate antibiotics (0.63, ie, the most optimistic estimate of effect from this data) then approximately 400 people would still need to be treated to prevent one admission.

The major effect on reducing reconsultations for persistent, new, or worsening symptoms with delayed antibiotics almost mirrors the results of the large DESCARTE (Decision rule for the Symptoms and Complications of Acute Red Throat in Everyday practice) cohort in sore throat, which helps support the likely validity of the findings.^26^ Although the estimates are compatible with the large GRACE trial in lower respiratory tract infection, the DESCARTE study found that prescribing immediate antibiotics did slightly reduce the number of reconsultations, unlike the findings in the current cohort. The slight difference between these studies might relate to inadequate control of confounding by indication, or alternatively adherence: adherence is probably considerably worse for chest infections in routine settings^13^ than in the context of trials,^4^ hence the antibiotic dose and duration may have been suboptimal for participants in the current study, resulting in more prolonged symptoms. For delayed prescriptions the delay advised was much shorter than that in the trials,^6^ hence, as in the DESCARTE cohort,^26^ it is likely that more than half will have used their delayed prescription. It is unclear how delayed prescriptions work in reducing the number of reconsultations, but the delay allows more time for patients to decide whether symptoms have resolved or progressed, and the delayed start means that the course will also finish later when symptoms are more likely to be settling, and hence patients will perceive less need to revisit the doctor.

### Conclusion

Prescribing immediate antibiotics may not reduce subsequent hospital admission or death for young people and adults with uncomplicated lower respiratory tract infection, and such events are uncommon. If clinicians are considering antibiotics, a delayed prescription may be preferable since it is associated with reduced number of reconsultations with worsening illness.

**What is already known on this topic**

A Cochrane review identified trials showing modest impact of antibiotics on short term resolution of symptoms of lower respiratory tract infection but was unable to document major adverse outcomes
Two studies using the UK General Practice Research Database (GPRD) suggest that the risk of pneumonia might be reduced by antibiotic prescribing
Most pneumonias can be readily treated in community settings without hospital admission, but neither GPRD study recorded the impact on major adverse outcomes such as hospital admission or death, nor could they control for confounding by indication

**What this study adds**

After controlling for the clinical propensity to prescribe antibiotics, it was not possible to show that prescribing immediate or delayed antibiotics reduced the major adverse outcomes of subsequent hospital admission or death
Such events occur less than 1% of the time, and even using the most optimistic estimate of benefit, 400 patients would have to be treated to prevent one death or admission
Patients commonly experienced non-resolving or new symptoms, and if clinicians are considering antibiotics a delayed prescription may be preferable, since unlike immediate antibiotics it is likely to be associated with statistically significant 36% reduction in this outcome

## Web extra

Extra material supplied by authors

**Supplementary information:** appendix 1

**Supplementary figure:** participant flow diagram

## Notes

We thank Jan Charlwood for discussing and agreeing the main focus of the programme; Terry Hamer for early input into the protocols, commenting on patient materials, and suggesting key outcomes; Samantha Richards-Hall for commenting on materials and participating in regular management meetings for overseeing the programme; the local general practitioners who promoted the study; and the doctors, practices, and patients who participated.

Contributors: SS developed the protocol; provided day to day overall management of the study; coordinated recruitment, follow-up, and data entry; and commented on drafts of the paper. PL had the original idea for the protocol, led the funding application, supervised the running of the study, contributed to the analysis, led the drafting of the paper, and is the guarantor for the paper. DM had the original idea for the study, led the original development of the protocol for the funding application, led the further protocol development and ethics submission, provided overall supervision of the study, and contributed to the analysis and the drafting of the paper. MM developed the protocol for funding and contributed to the management of the study, developing the detailed plan for analysis, and the drafting of the paper. MJT, with SS and DM, led the development of the protocol for ethical approval, supervised the study, and contributed to the analysis and drafting of the paper. KK, AvdB, and ML helped develop the protocol and contributed to developing the detailed plan for analysis, the analysis, and the drafting of the paper. BS developed the analysis protocol and led the quantitative analysis with DM and PL, and drafted the paper with PL.

Ethical approval: This study was approved by the Oxfordshire research ethics committee (A 09/HO604/67).

Data sharing: No additional data available.

Transparency: The manuscript’s guarantor (PL) affirms that the manuscript is an honest, accurate, and transparent account of the study being reported; that no important aspects of the study have been omitted; and that any discrepancies from the study as planned (and, if relevant, registered) have been explained
