# Supplementary material for: Antibiotic prescription strategies and adverse outcome for uncomplicated lower respiratory tract infections: prospective cough complication cohort (3C) study
Source: BMJ. 2017 May 23;357:j2148. doi: 10.1136/bmj.j2148 (PMC5439222; doi:10.1136/bmj.j2148)
Supplement: Supplementary file 1 — Supplementary information: appendix 1 [file litp035286.ww1.pdf]

Appendix 1

1) Comparison of characteristics from higher (higher than the median of 51 patients per practice) and lower recruiting practices

	High recruiting practices (n=131 practices)	Lower recruiting practices
**Age 60+ years**	8038/21711 (37.0%)	2805/7068 (39.7%)
**Female**	12924/21708 (59.5%)	4142/7066 (58.6%)
**Duration illness <7 days**	10485/21711 (48.3%)	3473/7068 (49.1%)
**Received pneumovax <10y**	4013/21711 (18.5%)	1260/7068 (17.8%)
**Ever smoked**	11482/21388 (53.7%)	3636/6923 (52.5%)
**Any co-morbidity**	9766/21711 (45.0%)	3294/7068 (46.6%)
**Lung co-morbidity**	5684/21711 (26.2%)	1753/7068 (24.8%)
**On steroids or bronchodilators**	5011/20907 (24.0%)	1500/6563 (22.9%)
**Living in top decile deprivation area** (most deprived*)	4392/21711 (20.0%)	1347/7068 (19.1%)
Symptoms		
**Shortness of breath**	14103/21668 (65.1%)	4345/6993 (62.1%)
**Fever**	8204/21684 (37.8%)	2745/7028 (39.1%)
**Chills**	6906/21676 (31.9%)	2217/7025 (31.6%)
**Chest pain**	8065/21686 (37.2%)	2547/7022 (36.3%)
**Confusion**	1450/21702 (6.7%)	406/7059 (5.8%)
**Coryza**	11938/21672 (55.1%)	3754/7010 (53.6%)
**Headache**	10030/21678 (46.3%)	3192/7017 (45.5%)
**Muscle aches**	7952/21675 (36.7%)	2519/7023 (35.8%)
**Diarrhoea**	1916/21705 (8.8%)	586/7049 (8.3%)
**Sputum: purulent**	13743/21709 (63.3%)	4444/7066 (62.9%)
**bloody/rusty**	749/21709 (3.5%)	360/7066 (3.7%)
Clinical examination		
**Severity assessment ≥ 5/10**	8480/21711 (39.1%)	3378/7068 (47.8%)
**Resp rate ≥ 24/min**	2058/21620 (9.5%)	812/7042 (11.5%)
**Temp ≥ 37.8°C**	1238/21706 (5.7%)	405/7052 (5.7%)
**Pulse ≥ 100/min**	2126/21703 (9.8%)	662/7064 (9.4%)
**O_2 sat < 95%**	1209/17696 (6.7%)	480/5722 (8.4%)
**SBP≤ 90 or DBP ≤ 60 mmHg**	1651/21711 (7.6%)	537/7068 (7.6%)
**Crackles**	9355/21706 (43.1%)	2854/7065 (40.4%)
**Bronchial breathing**	1512/21703 (7.0%)	644/7063 (9.1%)
**Wheeze**	5295/21704 (24.4%)	1753/7065 (24.8%)

2) Assessment of covariate balance in the stratified propensity scores.

Covariate balance was assessed by examining the standardised mean differences and a difference of 0.10 or more taken to indicate substantial imbalance (Normand ST, Landrum M, Guadagnoli E, et al. Validating recommendations for coronary angiography following acute myocardial infarction in the elderly: a matched analysis using propensity scores. J Clin Epidemiol. 2001 Apr;54(4):387-98.) Our main concern was the impact of residual confounding for immediate antibiotics. The table below shows that for immediate antibiotics, adequate covariate balance was obtained and if there was residual confounding, it is more likely to come from unmeasured confounders. With delayed prescribing, the covariate balance for most but not all covariates improved but there was still a risk of residual confounding (so it is possible we have underestimated the impact of delayed prescription on complications). It was not possibly to improve covariate balance further by increasing the number of strata or using another propensity score method (e.g. inverse probability weighting), although we did try this.

	**No antibiotics compared to Immediate antibiotics**		**No antibiotics compared to Delayed antibiotics**	
	**Standardised mean difference before propensity score stratification**	**Standardised mean difference after propensity score stratification**	**Standardised mean difference before propensity score stratification**	**Standardised mean difference after propensity score stratification**
**Age 60+ years**	0.288	0.066	0.086	0.011
**Female**	-0.055	-0.020	-0.032	0.039
**Received pneumovax <10y**	0.168	0.027	0.045	0.045
**Ever smoked**	0.114	0.007	0.006	0.019
**Lung co-morbidity**	0.214	0.022	0.065	0.047
**On steroids or bronchodilators**	0.221	0.020	0.058	0.065
Symptoms				
**Shortness of breath**	0.357	0.025	0.142	0.061
**Fever**	0.282	0.006	0.186	-0.076
**Chills**	0.235	0.041	0.143	0.007
**Chest pain**	0.070	-0.000	0.041	0.018
**Confusion**	0.066	-0.010	0.017	-0.025
**Coryza**	0.002	-0.002	0.097	-0.096
**Headache**	0.108	-0.001	0.127	-0.077
**Muscle aches**	0.140	-0.001	0.064	0.003
**Diarrhoea**	0.063	0.013	0.054	-0.004
**Sputum colour**	0.450	0.016	0.344	-0.093
Clinical examination				
**Severity assessment ≥ 5/10**	1.055	-0.007	0.425	0.113
**Resp rate ≥ 24/min**	0.226	0.029	0.031	0.133
**Temp ≥ 37.8°C**	0.197	0.013	0.055	0.052
**Pulse ≥ 100/min**	0.199	0.010	0.049	0.070
**O_2 sat < 95%**	0.297	0.020	0.061	0.012
**SBP≤ 90 or DBP ≤ 60 mmHg**	-0.057	-0.039	-0.084	-0.042
**Crackles**	1.782	0.041	0.652	0.047
**Bronchial breathing**	0.396	0.022	0.165	0.055
**Wheeze**	0.661	0.040	0.181	0.132

Summary of missing data

Variables	No missing (n=28 883)	Proportion (%) data missing
Reconsultation <30 days	0	0
Hospital admission or death	24	0.1
Antibiotic strategy	0	0
Age	0	0
Sex	5	0.02
Illness duration	0	0
Received pneumovax	0	0
Smoking status	469	1.6
Any comorbidity	0	0
Lung comorbidity	0	0
Steroid or bronchodilator use	1313	4.6
Deprivation	0	0
Symptoms:		
Shortness of breath	119	0.4
Fever	68	0.2
Chills	79	0.3
Chest pain	72	0.3
Confusion	18	0.1
Coryza	98	0.3
Headache	85	0.3
Muscle aches	82	0.3
Diarrhoea	26	0.1
Sputum colour **[Author: Purulent sputum?]**	4	0.01
Clinical examination:		
Severity assessment	10	0.03
Resp rate	119	0.4
Temp	21	0.7
Pulse	12	0.4
Oxygen saturation	5100	17.7
Blood pressure	34	0.1
Crackles	8	0.03
Bronchial breathing	13	0.1
Wheeze	10	0.01

Sensitivity analysis for missing data on oxygen saturation

Antibiotic prescribing	Reconsultation				Hospital admission			
	Multivariable risk ratio using stratified propensity score—assuming	P value	Multivariable risk ratio using stratified propensity score—assuming	P value	Multivariable risk ratio using stratified propensity score—assuming	P value	Multivariable risk ratio using stratified propensity score—assuming	P value

	**all missing values are oxygen saturation <95%**		**all missing values are oxygen saturation >95%**		**all missing values are oxygen saturation <95%**		**all missing values are oxygen saturation >95%**	
None	1.00		1.00		1.00		1.00	
Immediate	0.99 (0.92 to 1.07)	0.74	0.98 (0.91 to 1.06)	0.66	1.11 (0.67 to 1.84)	0.69	1.05 (0.64 to 1.75)	0.82
Delayed	0.64 (0.59 to 0.73)	<0.01	0.65 (0.59 to 0.72)	<0.01	0.85 (0.43 to 1.66)	0.63	0.80 (0.41 to 1.57)	0.52
