# Supplementary figures and images for: Antibiotic prescription strategies and adverse outcome for uncomplicated lower respiratory tract infections: prospective cough complication cohort (3C) study
Source: BMJ. 2017 May 23;357:j2148. doi: 10.1136/bmj.j2148 (PMC5439222; doi:10.1136/bmj.j2148)

# Figure 1: Participant flow diagram

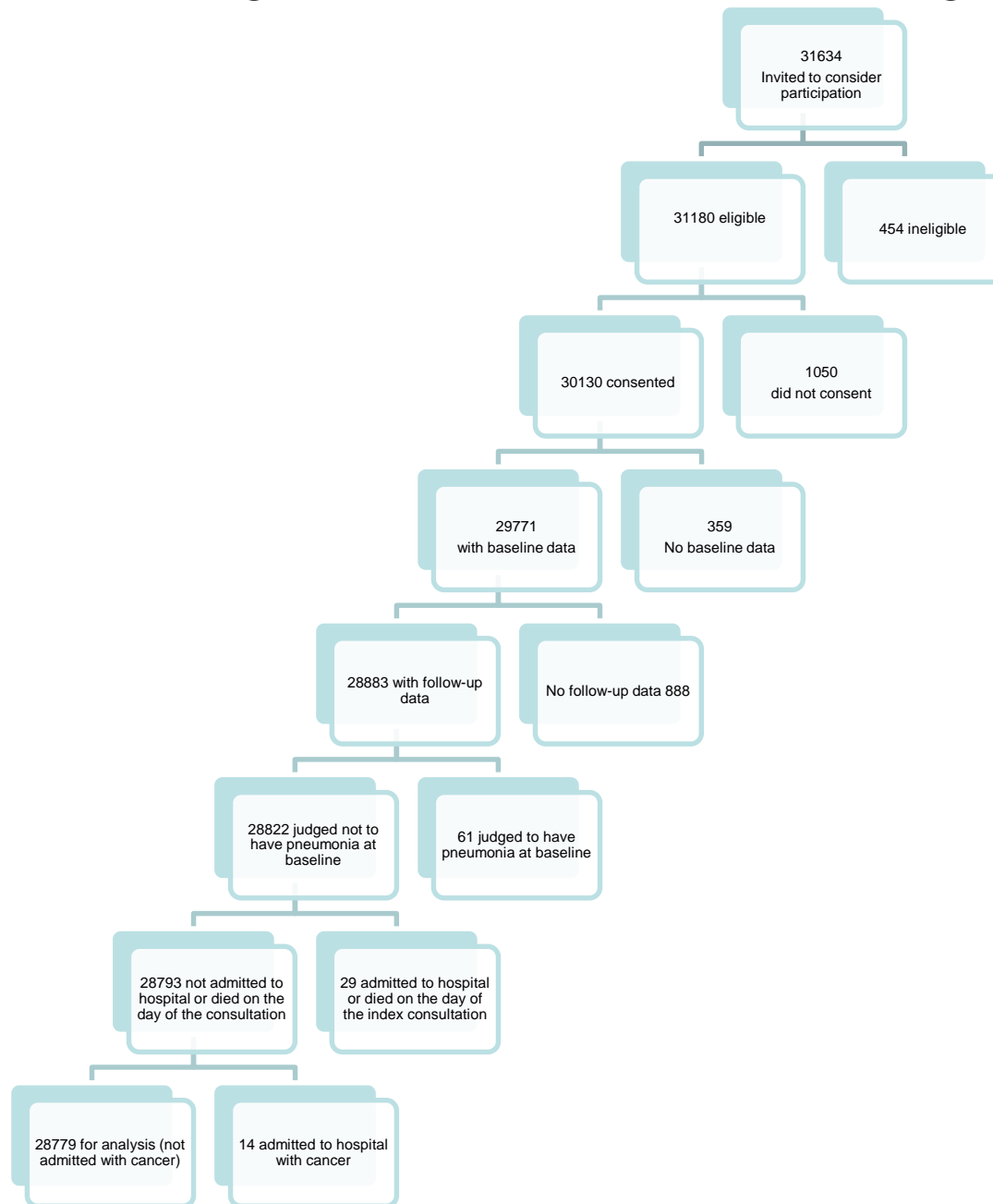

Supplement: Supplementary file 2 — Supplementary figure: participant flow diagram [file litp035286.wf1.pdf]
